# The gamma-glutamyl transpeptidase to platelet ratio (GPR) shows poor correlation with transient elastography measurements of liver fibrosis in HIV-positive patients with chronic hepatitis B in West Africa. Response to: ‘The gamma-glutamyl transpeptidase to platelet ratio (GPR) predicts significant liver fibrosis and cirrhosis in patients with chronic HBV infection in West Africa’ by Lemoine *et al*

**DOI:** 10.1136/gutjnl-2015-311133

**Published:** 2016-01-04

**Authors:** Alexander J Stockdale, Richard Odame Phillips, Anna Maria Geretti

**Affiliations:** 1 Department of Clinical Infection, Microbiology and Immunology, Institute of Infection & Global Health, University of Liverpool, Liverpool, UK; 2 Department of Medicine, Kwame Nkrumah University of Science & Technology, Kumasi, Ghana; 3 Komfo Anokye Teaching Hospital, Kumasi, Ghana

**Keywords:** HEPATITIS B, HIV/AIDS, FIBROSIS, LIVER CIRRHOSIS

Lemoine *et al*'s excellent article from the Gambia reported on non-invasive markers of liver fibrosis in patients with chronic HBV infection.^1^ They propose a novel biomarker—the gamma-glutamyl transpeptidase (GGT) to platelet ratio (GPR)—as a routinely available test that could identify patients with fibrosis or cirrhosis in resource-limited settings, thereby informing prognosis and guiding monitoring. Notably, the study excluded patients with conditions that might predispose to altered GGT or platelet counts, including pregnancy, significant alcohol consumption, use of antiviral therapy, acute malaria and hepatitis C, hepatitis delta or HIV coinfection. In their letter, Boyd and colleagues subsequently reported that in a French HBV/HIV coinfected cohort, GPR showed reasonable performance for identifying significant liver fibrosis.^2^

HBV and HIV are highly co-endemic in West Africa, and chronic liver disease is an emerging threat to the long-term health of HIV-positive patients in this region.^3^ Since 2010, we have been following a prospective cohort of patients attending the HIV clinic at the Komfo Anokye Teaching Hospital in Kumasi, Ghana, where prevalence of HBV co-infection is 14% (95% CI 12.4% to 15.8%).^4^ To date, 122 patients have undergone laboratory investigations paired with a valid measurement of liver fibrosis by transient elastography (TE) (Fibroscan F402, Echosens, Paris). Here, we report on the performance of the GPR and AST to platelet ratio index (APRI) in this cohort, in relation to TE measurements as a reference standard. Interpretive cut-offs were 7.6 kPa (F3: advanced fibrosis) and 9.4 kPa (F4: cirrhosis) as previously determined for HBV/HIV coinfection.^4^

For the purpose of the analysis, we excluded patients with pregnancy (n=2), significant alcohol consumption (n=1), acute malaria (n=2) and detectable HCV RNA (n=0) or hepatitis delta virus (HDV) RNA (n=1). In order to reflect the currently predominant profile of HIV-positive patients across sub-Saharan Africa, we only included patients established on antiretroviral therapy (ART) with two nucleoside/nucleotide reverse transcriptase inhibitors plus efavirenz (n=89) or nevirapine (n=11), and excluded those not receiving ART (n=6) or receiving protease inhibitors (n=10).

**Figure 1:**
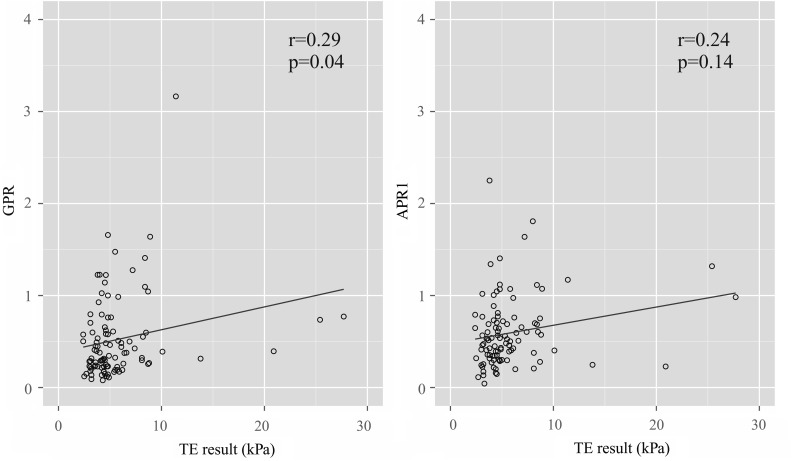
Association between AST to platelet ratio index (APRI) and GGT to platelet ratio (GPR) scores and transient elastography (TE) measurements. Bivariate correlations determined using Spearman's r. Linear association is represented by straight line.

The 100 patients (67% female) were median 44 years old (IQR 38–48), had received ART for median 7.2 years (5.0–9.0) and had a CD4 count of median 572 cells/mm^3^ (361–711); 78% showed plasma HIV-1 RNA suppression (<40 copies/mL). Median AST, GGT and platelet counts were 32 U/L (25–39), 60 U/L (44–81) and 165×10^9^/L (118–225), respectively. TE measurements were median 4.6 kPa (3.8–6.2). Of 100 patients, 18% and 7% were classified as having advanced fibrosis and cirrhosis, respectively. Factors significantly associated with TE measurements were GGT (r=0.33, p=0.001), AST (r=0.34, p=0.001), CD4 count (r=−0.21, p=0.033), APRI (r=0.24, p=0.014), GPR (r=0.29, p=0.004) (Spearman's r) and male gender (p=0.013, Mann–Whitney) (figure 1); there was no correlation with duration of ART (p=0.99) or age (p=0.76).

GPR and APRI had good negative predictive values for excluding cirrhosis (96%) and, to a lesser extent, advanced fibrosis (89% and 90%, respectively) (table 1). Overall diagnostic performance was poor for identifying advanced fibrosis and cirrhosis, however, with positive predictive values of 32% and 17% for GPR, and 29% and 14% for APRI. GPR had a better diagnostic performance than APRI for advanced fibrosis but not for cirrhosis (p=0.029 and 0.13, respectively, DeLong). It is important to note that the APRI threshold for the diagnosis of cirrhosis (>2.0) recommended by the WHO was unsuitable in this population, such that none of the seven patients with cirrhosis were correctly identified.^3^

**Table 1:** Performance of GGT to platelet ratio (GPR) and AST to platelet ratio index (APRI) compared with transient elastography (TE) as reference standard for the diagnosis of advanced fibrosis and cirrhosis in HBV/HIV coinfected patients in West Africa*

	Advanced fibrosis (TE threshold 7.6 kPa)	Cirrhosis (TE threshold 9.4 kPa)
GPR		
AUROC (95% CI)	0.73 (0.61 to 0.85)	0.71 (0.55 to 0.86)
Cut-off values	0.54	0.72
Sensitivity (%)	61	57
Specificity (%)	72	79
Correctly classified (%)	66	77
PPV (%)	32	17
NPV (%)	89	96
Positive LR	2.2	2.7
Negative LR	0.5	0.5
APRI		
AUROC (95% CI)	0.62 (0.46 to 0.78)	0.59 (0.32 to 0.85)
Cut-off values	0.57	0.70
Sensitivity (%)	67	57
Specificity (%)	63	74
Correctly classified (%)	64	73
PPV (%)	29	14
NPV (%)	90	96
Positive LR	1.8	2.2
Negative LR	0.5	0.6
APRI (WHO threshold)†		
Cut-off values	–	2.0
Sensitivity (%)	–	0
Specificity (%)		99
Correctly classified (%)	–	92
PPV (%)	–	0
NPV (%)		93
Positive LR	–	0
Negative LR		1.0

*Based on histologically defined interpretive cut-offs for patients with HIV/HBV coinfection as previously described.^4^

†As recommended in the “Guidelines for the prevention, care and treatment of persons with chronic hepatitis B infection (March 2015)”.^3^

AUROC, area under receiver operating curve; CI, confidence interval; LR, likelihood ratio; NPV, negative predictive value; PPV, positive predictive value.

The non-invasive and routinely available biomarkers GPR and APRI cannot be recommended for the diagnosis of advanced fibrosis and cirrhosis in HIV/HBV coinfected population established on ART in West Africa due to insufficient positive predictive value.
